# Beyond Cognitive Load Theory: Why Learning Needs More than Memory Management

**DOI:** 10.3390/brainsci16010109

**Published:** 2026-01-19

**Authors:** Andrew Sortwell, Evgenia Gkintoni, Jesús Díaz-García, Peter Ellerton, Ricardo Ferraz, Gregory Hine

**Affiliations:** 1 School of Education, The University of Notre-Dame Australia, Sydney 2007, Australia; 2 School of Health Sciences, The University of Notre Dame Australia, Fremantle 6160, Australia; 3 Research Centre in Sports, Health and Human Development, University of Beira Interior, 6201-001 Covilhã, Portugal; ricardo.ferraz@ubi.pt; 4 Department of Psychiatry, University General Hospital of Patras, 26504 Patras, Greece; evigintoni@upatras.gr; 5 Lab of Public Health, Epidemiology and Quality of Life, Department of Medicine, University of Patras, 26334 Patras, Greece; 6 Department of Psychology, University G’ d Annunzio, 66100 Chieti, Italy; jdiaz@unex.es; 7 BIND-Behavioral Imaging and Neural Dynamics Center, University G’ d Annunzio, 66100 Chieti, Italy; 8 Critical Thinking Project, School of Historical and Philosophical Inquiry, University of Queensland, Brisbane 4072, Australia; peter.ellerton@uq.edu.au; 9 School of Education, The University of Notre-Dame Australia, Fremantle 6160, Australia; gregory.hine@nd.edu.au

**Keywords:** neurobiology, science for learning, metacognition, cognitive reserve, brain endurance training

## Abstract

Background: The role of cognitive load theory (CLT) in understanding effective pedagogy has received increased attention in the fields of education and psychology in recent years. A considerable amount of literature has been published on the CLT construct as foundational guidance for instructional design by focusing on managing cognitive load in working memory to enhance learning outcomes. However, recent neuroscientific findings and practical critiques suggest that CLT’s emphasis on content-focused instruction and cognitive efficiency may overlook the complexity of human learning. Methods: This conceptual paper synthesises evidence from cognitive science, developmental psychology, neuroscience, health sciences and educational research to examine the scope conditions and limitations of CLT when applied as a general framework for K–12 learning. One of the major theoretical issues identified is the lack of consideration for the broad set of interpersonal and self-management skills, creating potential limitations for real-world educational contexts, where social-emotional and self-regulatory abilities are as crucial as cognitive competencies. Results: As a result of the critique, this paper introduces the Neurodevelopmental Informed Holistic Learning and Development Framework as a neuroscience-informed construct that integrates cognitive, emotional, and interpersonal dimensions essential for effective learning. Conclusions: In recognising the limitations of CLT, the paper offers practitioners contemporary, neurodevelopmentally informed insights that extend beyond cognitive efficiency alone and better reflect the multidimensional nature of real-world learning.

## 1. Introduction

Since its introduction by Sweller in the 1980s, Cognitive Load Theory (CLT) has been recognised as a potential construct in the field of instructional design in Australia and across Europe, primarily through its emphasis on managing working memory constraints to prevent cognitive overload [1]. Within the CLT construct, cognitive load is categorised into intrinsic, extraneous, and germane types. This categorisation provides a framework for structuring instructional material to facilitate efficient schemata (i.e., units of understanding) acquisition. According to CLT, intrinsic cognitive load is claimed to refer to the inherent complexity of the learning material itself and how many elements must be processed simultaneously in working memory. Extraneous cognitive load is described as the unnecessary strain placed on learners by poor instructional design or superfluous information that does not directly contribute to learning. Germane cognitive load is considered the beneficial mental effort devoted to reorganising and integrating new information into existing knowledge structures, ultimately enhancing learning and retention. The assumptions of CLT also range across the conceptualisation of knowledge as biologically primary (innate) or secondary (teachable). These working memory and knowledge categories are, however, contentious and not well supported by evidence [2,3,4], making consequent claims as to the mechanisms of learning optimisation dubious.

Importantly, CLT was not proposed initially as a general theory of education or human development. Instead, it emerged from late-twentieth-century cognitivist psychology as a framework for optimising instructional efficiency under constrained conditions, particularly for novice learners acquiring well-structured knowledge. Recent philosophical and neuroscientific analyses further suggest that CLT has undergone substantial modification over time, raising questions about whether these changes represent cumulative theoretical refinement or compensatory extension in response to unresolved empirical challenges [4]. In recent educational discourse, however, CLT is increasingly positioned as a broadly applicable pedagogical framework for K–12 education [5]. It is this extension beyond CLT’s original scope, rather than the theory’s foundational contributions, that motivates the present critique.

Proponents of CLT, nonetheless, assert that this framework positively influences guided content-focused instruction. We do not dispute this contribution. Instead, the present paper argues that difficulties arise when CLT-based instructional prescriptions are treated as universally applicable across diverse learners, developmental stages, and educational contexts. It is the focus of this paper to provide a better representation of current knowledge in the neuroscience of learning and a deeper understanding of student learning across a broader range of experiences than what is or can be accommodated by CLT. This understanding includes non-cognitive dimensions of learning, such as the development of interpersonal and self-regulatory competencies that recent neuroscience and educational psychology insights suggest are crucial for long-term learning, personal development, and real-world success [6,7]. Indeed, CLT continues to drift further from current innovative models of learning that focus on developing autonomy and fostering personal and social capabilities. Moreover, while CLT offers a parsimonious account of instructional efficiency, its explanatory scope is largely confined to memory management, limiting its capacity to account for learning processes that unfold across emotional, social, and developmental contexts.

Neuroscientific evidence increasingly supports that cognitive and emotional processes involved in learning are highly interrelated. For instance, neuroimaging data reveal activity in brain structures related to emotion, including the amygdala and prefrontal cortex, during learning and remembering [8]. In this respect, emotionally salient experiences are better remembered than neutral experiences, suggesting that emotional engagement may facilitate learning by affecting attention and memory consolidation [9]. Moreover, the prefrontal cortex, a critical region of the brain for executive functions (i.e., memory and cognitive control) and CLT, is similarly important for emotional regulation and social cognition [10]. An implication of this is that the cognitive processes that CLT emphasises are closely intertwined with emotional and social factors that the theory largely neglects. Therefore, the CLT construct appears to be deficient in accounting for learning that involves the art of managing overwhelming emotions, motivation, and social interaction factors.

Accordingly, the present paper does not focus only on the accuracy of CLT but also on its incompleteness when applied as a dominant explanatory framework for K–12 learning. We examine, for example, that several commonly invoked assumptions and inferences sit uneasily with contemporary evidence from neuroscience and developmental psychology, particularly when learning is conceptualised as a dynamic, adaptive process unfolding across childhood and adolescence. The breadth of constructs addressed is intentional and reflects the multidimensional nature of learning, which cannot be fully captured by cognitive load considerations alone. This paper proceeds as follows: we first explore how CLT’s assumptions conflict with neuroscientific findings, before examining the critical importance of early brain development, emotional and social factors, and self-regulation. Finally, we propose a new conceptual, hypothesis-generating framework to support a more comprehensive understanding of learning, arguing that a more comprehensive approach could better reflect the realities of cognitive and emotional development across diverse educational settings.

## 2. Cognitive and Brain Reserve Development in Early Life

Cognitive reserve refers to the mind’s ability to optimise or maximise performance in the face of brain aging or pathology, while brain reserve refers to the physical aspects of the brain that contribute to cognitive function, such as the volume of neurons and synapses [11]. Both reserves are shaped significantly during early life through various factors, including educational experiences, social interactions, and environmental enrichment (e.g., training interventions) [12,13].

Neuroscientific evidence indicates that enriched environments, characterised by stimulating cognitive and social experiences during formative years, enhance brain development and increase cognitive reserve [12,14,15,16]. Enriched environments have been associated with increased neurogenesis (i.e., the birth of new neurons); synaptogenesis (i.e., the formation of new connections between neurons); and dendritic branching, which is the growth of neuronal extensions that receive signals within the hippocampus, a brain region strongly involved in learning and memory formation [12,14,15,16]. Physically active learnings, an example of an enriched and combined cognitive and physical environment, has been shown to improve neuroplasticity and cognitive capacities among children [17,18]. While these findings do not prescribe specific instructional methods, they collectively suggest that learning capacity is malleable and responsive to sustained engagement and challenge rather than solely constrained by fixed limits.

Children’s remarkable learning capabilities are facilitated by a large brain that offers the neural framework for language, dynamic working-memory capacity, concentrated attention, self-regulation, and planning; furthermore, children exhibit a range of evolved adaptations that enable them to uniquely adjust to different cultures, environments and context [19]. These evolved adaptations include fundamental perceptual and cognitive mechanisms that are elaborated throughout development, a high level of plasticity (i.e., an individual’s ability to alter behaviour, cognition, and neural structure), exceptional social-learning abilities and inclinations for exploration and play [19,20]. Accordingly, developmentally appropriate challenge, variability, and engagement should be understood as core mechanisms of learning that underpin the development of robust cognitive and neural reserves. These serve as the foundation for children’s remarkable learning capacities and offer educators valuable insights into optimal educational approaches for children and young people [21]. Therefore, providing school students with stimulating, enriched learning environments and student-centred activities is highly beneficial for promoting the development of long-term cognitive health and adaptability.

While CLT can remind teachers to consider students’ capacity to effectively engage with the content to be taught, regulating student cognitive load repeatedly through the CLT encouraged structured lesson activities can result in a sterile learning environment. For example, some CLT-aligned implementations, particularly those that prioritise minimising difficulty and tightly controlling task structure, may inadvertently narrow the range of learning experiences available to students. This sterility may reduce student engagement, motivation, and opportunities for student-centred activities, thus impairing the potential for cognitive and brain reserve development. Previous research has established that emotional and social factors are essential for learning, suggesting that overly simplistic or unengaging environments could hinder the development of critical thinking and problem-solving skills [7,22,23,24]. Therefore, creating an environment that supports students to become agents of their learning, such as developing students’ ability to self-assess and decide their own progress, is one of the most impactful processes in student learning and maturity development [25,26].

Children exposed to various learning opportunities, particularly those that promote critical thinking and problem-solving and offer a degree of challenge, are more likely to develop a robust cognitive reserve to manage cognitive loads better later in life [12,27,28]. To complement prior, cross-sectional research on this topic, two longitudinal studies have also shown that early development of self-regulation abilities not only contributes to cognitive and learning success in childhood but also has lasting impacts across the lifespan, influencing health, substance addiction, criminal behaviour, educational achievement, income, financial management, pregnancy planning, and parenting style [29,30]. Thus, early cognitive and self-regulatory development throughout childhood can influence academic outcomes but also broader life trajectories.

Children’s pronounced tendency to explore is crucial to their remarkable learning abilities, especially as their level of maturation is a time of heightened plasticity [29,31]. This heightened neuroplasticity and dynamic patterns of brain development characteristic of childhood represents a uniquely fertile period for learning and cognitive growth. Changes in grey and white matter in the brain, which characterise growth and development during childhood and adolescence, indicate the potential significance of cognitive and brain reserve development in these stages. Accompanying skill-development (i.e., acquisition and strengthening of cognitive, emotional, and self-regulatory abilities) can create a greater cognitive reserve among subjects with positive effects for them in the long-term, such as their cognitive health-span throughout their life [32]. Importantly, these developmental advantages extend beyond childhood, as enhanced cognitive reserve acquired early in life has been associated with improved cognitive functioning and resilience in older adulthood [33].

The aforementioned brain changes are associated with alterations in physical and emotional behaviour [34]. During this heightened period of plasticity, children also have a heightened ability to effectively discern causal patterns in their environments [14,15,35,36]. Furthermore, children can exhibit decision-making superior to adults when utilising probabilistic information [37], demonstrating greater efficiency (i.e., shorter durations) in solving problems that necessitate unconventional use of objects (i.e., turning a learning activity into a game-like experience) [37,38,39]. These results are in line with previous evidence suggesting that combined training induces significant cognitive benefits in youths compared to adults [40,41]. Moreover, it appears that younger children possess more accurate memory performance in specific contexts (i.e., verbatim memory) than adults [42,43]. These noted characteristics of the child and adolescent learner have important implications for the individual relating to the concept of cognitive and brain reserve, which suggests that an enriched environment and early-life learning experiences can enhance neural efficiency and adaptive cognitive functioning.

Based on the science for learning, we argue that CLT’s narrow focus on reducing cognitive load might inadvertently overlook the importance of fostering enriching educational experiences, potentially impeding the development of cognitive and brain reserves and self-regulatory and executive skills vital for lifelong learning. The encompassing universalistic pedagogical instructional approach that CLT promotes does not account for a classroom of learners’ with diverse learning needs, backgrounds and experiences [44]. For instance, students with higher cognitive reserves may better manage extraneous and germane load, while those with lower reserves may struggle, leading to disparities in learning outcomes [45,46,47]. This variability supports the need for instructional designs that adapt to individual learners’ cognitive, self-regulatory capacities and experiential backgrounds rather than simply adhering to CLT principles.

## 3. Oversimplification of Working Memory and the Role of Prefrontal Cortex in Cognitive Capacity

The central issue is not whether working memory is limited, but whether CLT’s commonly invoked ‘bottleneck’ metaphor adequately reflects contemporary evidence showing that cognitive capacity is dynamically regulated, strategy-dependent, and developmentally malleable. One of the foundational critiques of CLT is its reliance on an overly simplified model of working memory, which assumes a relatively fixed-capacity system constrained by strict limits [48]. This generalisation has influenced instructional designs intended to prevent cognitive overload in an effort to support effective learning. Opposing this theory are neuroscientific findings that reject such rigidity and linearity. Current research shows that working memory arises out of fluid, distributed brain networks that shift adaptively based on the nature and demands of specific tasks [49]. Indeed, working memory operates across a distributed neural network that is both adaptable and context-sensitive [41,50,51,52,53]. Taken together, the research necessitates instructional designs that progressively stretch learners rather than prematurely constrain load [3].

Functional neuroimaging studies show that working memory tasks engage a broad frontoparietal network, including the dorsolateral prefrontal cortex, posterior parietal cortex, and anterior cingulate cortex, that operates in a context-dependent manner [54]. These findings suggest that cognitive capacity is not static but adaptive, shaped by experience, motivation, and self-regulatory processes. Depending on factors such as emotional significance, novelty of the task, and attentional load, various neural circuits are dynamically engaged to allow the brain to dynamically reconfigure resources to optimise performance. Thus, the problem with CLT framing working memory as a ‘rigid bottleneck’ is that it provides an inaccurate depiction that may lead to educators underestimating learners’ capacity for cognitive growth and adaptive expertise. By prioritising a primary focus on reducing cognitive load, traditional CLT-matched instruction may actually restrict potential growth of the learner’s cognitive abilities (i.e., cognitive flexibility, creativity, resilience) that are increasingly identified as critical throughout life [55]. As Shenhav [56] suggested, moderate cognitive difficulty, combined with emotional engagement and self-motivated control, can enhance neural plasticity, which then supports executive control mechanisms, creating stronger cognitive systems. Therefore, instructional designs could more effectively support learners by varying cognitive load dynamically, encouraging stretching and cumulative construction of working memory systems over time.

Brain Endurance Training, a novel training method that involves cognitive activities priming or intermixed with physical activity, has demonstrated the cognitive benefits of regular physical activity on working memory. While much of this evidence has been primarily focused on adults and the elderly, active learning, a form of combined cognitive and physical training, has also been positively correlated with greater academic and cognitive performance [57]. Studies show that working memory capacity can be modulated by factors like attentional focus and neural plasticity, particularly in task-relevant regions like the prefrontal cortex (PFC) and parietal lobes [49,58].

When faced with complex tasks, the brain recruits additional resources from regions like the dorsolateral PFC, indicating that cognitive capacity is dynamic rather than fixed [59]. By conceptualising working memory as a bottleneck, CLT may not fully capture this neural adaptability, particularly for tasks that require creative thinking, problem-solving, or adaptive responses. In addition, CLT’s primary focus on managing cognitive load for instructional efficiency may inadvertently discourage instructional practices that challenge working memory in ways that stimulate brain development and enhance cognitive reserve and resilience. For example, complex problem-solving tasks activate a broader neural network, including areas essential for cognitive growth and adaptability [60]. Related to this is the capacity of the brain to maintain performance when fatigued, which is understood as a psychobiological state of tiredness and impaired cognitive performance induced by cognitive and emotional demands of daily life. Limited cognitive inputs or trainings (as CLT implies) may fail in the development of the capacity to adapt responses under fatigue. Mental fatigue has been linked with higher feelings of exertion and greater PFC activity [61]; it is inherent in school time and can impair performance and learning capacity. While sufficient evidence exists to show that cognitive or combined trainings can improve the adaptative capacity of people to self-regulate their response in the presence of mental fatigue [41], CLT fails to account for this important development. Furthermore, this underscores the importance of instructional experiences that not only manage immediate task demands but also support the development of adaptive cognitive endurance, an area largely unaddressed within CLT.

Neuroscientific data indicate that working memory is neither an independent, rigid entity with fixed capacity nor an independent, modular system, but rather a dynamic and distributed network that adapts to task demands in a flexible way [41,50,51,52,53]. A review of functional magnetic resonance imaging (fMRI) studies on the performance of working memory tasks indicates the involvement of a widespread network that includes the PFC, parietal cortex, anterior cingulate cortex, and subcortical structures, such as the basal ganglia [49,58]. Specific regions that are involved and the extent of their activation depend on the nature of a task, type of information processed, and individual differences related to expertise or cognitive reserve. By promoting instructional designs that overly simplify content to reduce load, CLT may prevent students from engaging in cognitive tasks that promote long-term neural development, particularly in more demanding cognitive tasks.

## 4. Insufficient Focus on Interpersonal and Emotional Skills

Genetic predispositions (nature), environmental contexts (nurture), and the dynamic interactions between both these factors, including epigenetic mechanisms, fundamentally influence human development [62,63]. These elements are integral to learning processes and the reorganisation of neuronal networks that underlie the formation of neural representations for new knowledge. As previously mentioned, a significant limitation of CLT is its narrow focus on content delivery and schema development, which often sidelines developing interpersonal and emotional skills essential for learning in real-world settings. The acquisition and practice of novel knowledge or skills generate distinct and recurrent activity patterns within the brain. Furthermore, neuroscientific research has shown that social and emotional learning (SEL) is critical for academic success and life outcomes, as these skills activate neural pathways involved in empathy, emotional regulation, and cooperation [64,65]. For example, they help individuals understand and replicate the actions and emotions of others [66,67,68]. Importantly, SEL processes are not peripheral to cognition; rather, they directly influence attention, motivation, persistence, and the capacity to engage in complex learning tasks [69]. CLT’s exclusive focus on cognitive load neglects integrating instructional strategies that promote these social and emotional competencies.

The omission of interpersonal skills from CLT [3,4] is particularly problematic in collaborative learning environments, where students must process information while negotiating, communicating, and empathising with others. Consistent with the neuroscientific literature, student learning through social interactions strengthens neural networks associated with cognitive and affective processes, contributing to a more holistic learning experience [70,71]. Furthermore, the acquisition and practice of interpersonal skills generate distinct and recurrent activity patterns within the brain [72,73,74]. In contrast to contemporary neuroscientific research, teachers who focus narrowly on content applying CLT-based instructional design may fail to provide students with the social experiences necessary for developing empathy, teamwork, and other interpersonal skills essential for professional and individual personal success. A collaborative problem-solving task in which students analyse a real-world issue enhances conceptual understanding and cultivates communication, perspective-taking, and cooperative decision-making skills essential for academic success and interpersonal growth.

A recent systematic review by Blewitt [65] elucidated the neural underpinnings of SEL, showing that these skills in K–12 students are not just ‘soft skills’ but are deeply intertwined with cognitive and physiological processes and engage specific neural pathways involved in empathy, emotional regulation, cooperation, and social cognition. For example, the acquisition and practice of novel knowledge or skills, including social and emotional competencies, generate distinct and recurrent activity patterns within the brain, leading to structural and functional changes in the relevant neural networks [72,73,74]. Brain regions such as the medial prefrontal cortex, amygdala, insula, and superior temporal sulcus have been implicated in processing social and emotional information, as well as understanding other people’s mental states (i.e., theory of mind) and regulating one’s own emotions [66,67,68]. Mirror neurons are a class of special neurons that are fired when a person acts and when they observe somebody else acting, thus providing insight into others’ actions and emotions while helping them learn new things and mimicking people through observing actions and understanding actions [75]. These findings give reason to underscore the biological grounding of social and emotional learning and raise the need for integration of these aspects into educational practices.

Developmental psychology research, in conjunction with the neuroscientific literature, has shown that social interaction is a powerful force in cognitive development, particularly during childhood and adolescence [76,77]. Through meaningful interactions with caregivers, other students, and teachers, children develop the ability to control their emotions, interpret social signals, and conduct themselves appropriately in social hierarchies, gaining a sense of self within a social framework. These experiences advance the development of the brain regions specifically involved in social cognition, such as the prefrontal cortex, and also enhance the interconnectivity between these regions and other areas that support cognitive control, emotional processing, and memory [7,78].

## 5. Neglect of Self-Regulation and Executive Function Skills

Self-regulation and executive functions like planning, attention management, emotional regulation, updating working memory, and cognitive flexibility are increasingly considered the foundations of independent learning, psychological well-being, and long-term adaptation (i.e., resilience). These skills enable individuals not just to receive information but to plan, monitor progress, adjust strategy, and show grit through difficulty [79]. Based on research in the field of neuroscience, the development of executive function and maturation primarily involve the prefrontal cortex (PFC), while also depending on the broader network of brain regions (i.e., anterior cingulate cortex, basal ganglia, and parietal lobes) and their interconnections [80,81]. For example, the prefrontal cortex is the part of the brain region responsible for the regulation of higher-order cognitive operations [81], that engages and interacts with networks such as the anterior cingulate cortex, basal ganglia, and parietal lobes to make flexible goal-directed behaviour possible [82,83]. Furthermore, neuroplasticity research has shown that executive functions can be developed in learning environments through purposeful practice, which explicitly supports the development of self-monitoring (i.e., reflection logs), strategic decision making (i.e., problem-based learning), and emotional self-regulation (i.e., mindfulness training) [84,85].

Nurturing the development of self-regulation and executive functions in students fosters the evolution of a student as a metacognitive learner who can act in a mindful way, monitor their progress, flexibly adapt their strategies, and reflect on their learning. However, CLT, with the premise and prioritising of optimisation of cognitive efficiency when instructing, is less likely to provide adequate emphasis and focus on pedagogical strategies for building metacognitive and executive skills. By placing the mitigation of cognitive load at the center of knowledge acquisition, CLT-oriented approaches may deprive learners of the opportunity to exercise essential self-regulatory skills, such as managing ambiguity, managing frustration-evoked emotions, or coping with complex, open-ended tasks, which are essential for genuine problem-solving and adaptive behaviour [86]. Moreover, self-regulation and executive functions predict not just academic achievement but also lifelong mental health, vocational achievement, and social competence [30]. Instructional designs that deemphasise these factors risk producing students who excel in very structured settings but fail when given the demands of independent, lifelong learning. Thus, it is essential to nurture executive function growth through specially designed cognitive and emotional challenges to ready individuals for success in an ever-changing world.

Beyond interpersonal skills, CLT lacks emphasis on addressing the development of self-regulation and executive functioning skills, which are essential for managing one’s learning process, particularly in complex or high-stakes environments. Consistent with contemporary neuroscientific research, the prefrontal cortex plays an important role in executive functions (i.e., planning, goal-setting, attention management, and emotional control) which are critical for effective learning and decision-making [55,80]. Moreover, these skills enable students to more effectively manage their cognitive resources, prioritise tasks, and stay focused despite distractions, effectively optimising working memory, resulting in extending working memory capacity. For example, dopamine activity in the prefrontal cortex is key in regulating attention and managing working memory resources [87]. Students with stronger executive functions are better equipped to handle higher cognitive loads by strategically focusing their attention and filtering out distractions. Both benefits on cognition and associated-higher oxygenation on PFC have been found by engaging in Brain Endurance Training environments [88]. Therefore, a classroom that repeatedly aligns practices with CLT’s focus on instructional strategies that reduce cognitive load impedes student development of executive skills, which are essential for self-directed learning and resilience in challenging or unfamiliar contexts.

CLT’s lack of emphasis on self-regulation overlooks how learners can be taught to handle cognitive load independently. Training students in metacognitive strategies, such as self-monitoring, reflection, and adaptive problem-solving, can improve cognitive flexibility and resilience [89]. Neuroscientific evidence supports the role of metacognition in strengthening neural pathways associated with executive control, which promotes effective self-regulation in learning [90,91]. CLT’s content-centric approach may fail to cultivate those skills necessary to independently manage cognitive load, resulting in learners who may excel in structured tasks but struggle with independent or real-world challenges.

To date, several recent studies have reported neuroscientific evidence indicating the importance of metacognition and self-regulation for learning, supported by their neural underpinnings [92,93]. It has been shown that metacognitive activity is associated with increased activity of the PFC, especially the dorsolateral and medial prefrontal regions, along with the anterior cingulate cortex (ACC) [94,95]. These brain areas seem to be involved in executive functioning, such as the processes of planning, decision-making, working memory, and cognitive control, all indispensable components of the metacognitive process. Applying metacognitive strategies, such as self-monitoring and self-assessment, sharpens neural tracts related to executive control, with the result of better self-regulation when learning occurs [90,91]. Innovative fMRI studies have found that the medial PFC is more active in individuals who are better at estimating their performance on cognitive tasks, pointing toward a neural basis of metacognitive accuracy [94,96]. Accordingly, metacognition and its neural correlates in enhancing learning processes provide further support for integrating self-regulatory teaching and learning strategies to improve student cognitive performance and educational outcomes.

## 6. Challenges in Differentiating Cognitive Load Types

Empirical neuroscience findings reveal that cognitive processes are highly interdependent, challenging the rigid separation of intrinsic, extraneous, and germane loads [97]. These cognitive load categories are intended to differentiate between load arising from task complexity, instructional design, and learning-related cognitive effort, respectively. While this tripartite distinction has been influential in instructional research and design, its empirical and theoretical robustness has been increasingly questioned, particularly in light of findings from neuroscience and cognitive science demonstrating the interdependence of cognitive processes [5].

Authentic or real-world learning involves complex, fluid interactions among cognitive functions, highlighting the need for instructional approaches that reflect this natural integration. A limitation of CLT (noted earlier) is that it struggles to empirically distinguish intrinsic, extraneous, and germane cognitive loads. Cognitive processes are, in fact, highly interdependent in neural reality. Pessoa [98] suggested that brain regions responsible for processing various cognitive tasks often overlap, with networks working in a coordinated rather than isolated manner. This interconnectedness implies that cognitive load types are not easily separable and may vary significantly depending on individual contexts and learning conditions. For instance, fMRI studies show that brain activation for tasks associated with intrinsic or extraneous load often involves overlapping regions, particularly in the prefrontal cortex, challenging CLT’s rigid categorisation of cognitive load [99,100,101].

The lack of clarity around neural markers for different load types suggests that instructional strategies based on strict distinctions may be less effective in real-world scenarios, where cognitive demands as previously noted, are often fluid and context-dependent [102,103]. The difficulty in empirically distinguishing between load types complicates the practical application of CLT and suggests the need for a more flexible approach that can adapt to the natural integration of cognitive processes in the brain. Furthermore, it is problematic to empirically distinguish between different load types due to highly connected and overlapping cognitive processes in the human brain. Learning itself is a continuous and non-linear process that cannot be divided neatly into separate processing steps for intrinsic and extraneous load. Instead, it involves a complex interplay of various cognitive functions, including attention, perception, working memory, long-term memory, and executive control, all mediated by distributed and interacting neural networks. For example, the intrinsic load stemming from new information a learner is supposed to process always interacts with how that information is presented. The presentation might induce extraneous load when not properly designed. At the same time, the learner will also work cognitively on comprehending and integrating that information, which is germane load. Even if these categories are given to exist, they would not do so entirely separately; rather, they would overlap and dynamically interact with each other. These different types of loads will need to share limited attentional resources, and the strategy of allocation is likely to depend on a variety of factors, including the learner’s prior knowledge, motivation, and metacognitive skills.

## 7. Overemphasis on Extraneous Load Reduction

While CLT may be beneficial for certain types of learning, CLT’s emphasis on reducing extraneous load overlooks the potential cognitive benefits of moderate distractions or extraneous information. While CLT’s emphasis on reducing extraneous load can enhance efficiency, neuroscience research on the brain’s Default Mode Network (DMN) suggests that moderate distractions can foster creativity, cognitive flexibility, and problem-solving. Excessive simplification of learning environments may inadvertently suppress these adaptive skills, hindering deeper learning and engagement. The DMN has been studied extensively and shown to activate during periods of distraction or rest, playing a significant role in creative thinking, memory consolidation, and cognitive flexibility [104]. Moderate extraneous stimuli can activate areas such as the anterior cingulate cortex, enhancing attentional flexibility and enabling learners to better adapt to complex or changing environments [105,106].

More recent research has demonstrated that the DMN is integral to a range of cognitive processes important for learning and problem-solving. The DMN is responsible for consolidating newly learned information in long-term memory, usually during periods of rest or low cognitive load [107]. Furthermore, activity within the DMN has been associated with creative thinking, insight problem-solving, and novel idea generation [60]. It is considered that this may be achieved through spontaneous information recombination and the exploration of alternative perspectives. Additionally, the DMN might provide cognitive flexibility through which the brain may switch between various mental states and consider different perspectives easily, according to Vatansever [108]. In this regard, evidence of the involvement of the DMN in self-referential thinking, access to autobiographical memory, and mental simulation that provides important facilitation to a coherent ‘sense of self’ and the ability to learn from one’s experiences has been documented [109].

Research on attention and cognitive control has suggested that a moderate level of distraction or extraneous stimuli may enhance attentional flexibility and promote adaptive learning. The anterior cingulate cortex (ACC) is part of the limbic system and is involved in cognitive control and conflict monitoring; it is activated when the brain encounters distracting, unexpected or conflicting information [105,106]. Low levels of distraction can be positive, providing a type of ‘cognitive training’ improving performance of the ACC, by enhancing filtration and removal of irrelevant information and adapting to the fluctuating task demands, shifting between different attentional states. However, high levels of distraction may negatively influence performance. The development of increased attentional flexibility is very important for dealing with complex, unpredictable, and dynamic real-world environments where one has to adapt to and shift between different focuses of attention.

Educational psychology also offers some insight into the potential benefits of appropriately challenging learning experiences. For example, theories of motivation, such as self-determination theory, suggest that autonomy, competence, and relatedness are important in fostering student intrinsic motivation and engagement [110]. Learning environments that are overly simplified and lack challenge may fail to satisfy students’ basic psychological needs, leading to classroom boredom, disengagement, and a lack of deep learning. Moreover, the theory of ‘productive failure’ suggests that the struggle to solve well-designed, challenging problems even when initial efforts are not successful leads to deeper understanding and greater transfer of learning than does being presented with a worked example [111]. The potential cognitive benefits of moderate distractions, DMN activity, and challenging learning experiences thus suggest that an overemphasis on extraneous load reduction by traditional CLT advocates may be counterproductive in certain contexts. Educators who seek to minimise extraneous load, adhering to CLT-based instructional approaches, may create a learning environment that discourages experiences that foster adaptive thinking, creativity, and resilience skills critical for navigating multifaceted real-world situations. The psychologist Daniel Kahneman has also shown that striking improvements in cognitive tests occur (with error rates falling from 90% to 35%) when participants have been put under increased extraneous cognitive load. This is particularly noticeable when an intuitive but incorrect response to a problem is present [112].

Although the CLT’s emphasis on the management of cognitive load has significantly influenced instructional design, this strong emphasis on reducing extraneous load may be overly restrictive and arguably detrimental in some aspects of learning. Neuroscience evidence about the DMN and the role of moderate distractions, combined with insights from educational psychology on motivation and engagement, suggests that a more nuanced approach is needed [113]. Indeed, a balance needs to be achieved in managing cognitive load with the potential benefits of providing appropriately challenging and engaging learning experiences that foster creativity, cognitive flexibility, resilience, and the ability to adapt to the complexities of real-world situations. By embracing a more holistic view of cognitive load, one that incorporates the dynamic interplay between cognitive, emotional, and neural factors, educators can create learning environments that will optimise learning and better prepare students for success in a rapidly changing world.

## 8. Proposed Holistic Framework for K–12 Teaching and Learning

**Figure 1:**
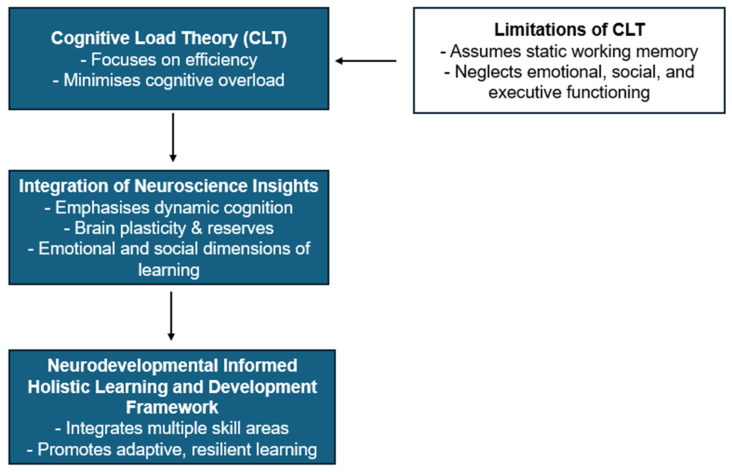
Theoretical framework: from cognitive load to neurodevelopmental informed, holistic learning and development.

**Figure 2:**
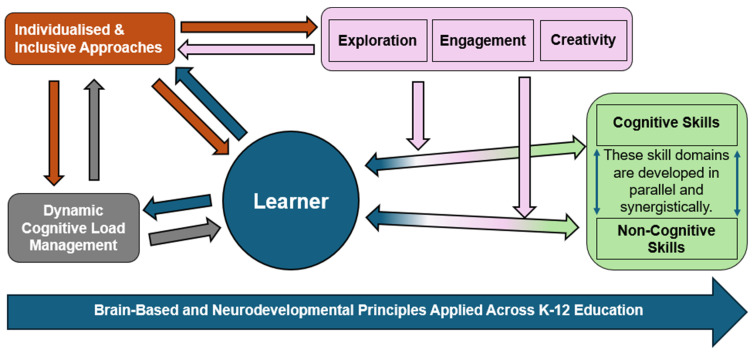
An illustrative neuroscience-based construct of distributed learning processes.

This paper proposes an innovative approach, the Neurodevelopmental Informed Holistic Learning and Development Framework (NIHLDF), as a conceptual, hypothesis-generating, illustrative construct, one among several [5,114,115], that draws upon recent advancements in neuroscience, emphasising that learning is a dynamic, distributed process across various neural networks (See Figure 1). We offer NIHLDF as a candidate solution that addresses the aforementioned problems (see Figure 2 for a graphical representation of the core components and relationships within the NIHLDF framework). NIHLDF reconceives cognitive load by rejecting the oversimplified view of it as a fixed capacity, instead viewing cognitive resources as adaptable and context sensitive. It also recognises the importance of cognitive and brain reserves in early childhood development, shaped by enriched environments, social interactions, and problem-solving activities.

The NIHLDF is not intended to replace existing theories such as self-regulated learning, social–emotional learning, or biopsychosocial models, but to integrate them within a neurodevelopmental framing that foregrounds dynamic cognitive demand, reserve development, and regulatory skill growth across K–12 education. The framework does not assert empirical confirmation; instead, it functions as a heuristic structure for organising existing evidence and generating testable hypotheses for future research. Its contribution lies in explicitly linking instructional design decisions to both knowledge acquisition and the development of adaptive self-regulatory and interpersonal capacities that support learning in varying contexts of complexity.

The NIHLDF is grounded in three central conclusions emerging from the preceding analysis. First, learning capacity is dynamic, developmentally contingent, and shaped by experience rather than determined solely by fixed working memory limits. Second, cognitive, executive, emotional, and social processes are functionally interdependent and cannot be meaningfully isolated in authentic learning environments. Third, instructional approaches that prioritise short-term efficiency through load reduction provide an incomplete account of how learners develop the capacity to manage complexity, persist through challenge, and adapt across contexts and over time.

Importantly, the relationships depicted in Figure 2 are conceptual and reciprocal rather than causal or sequential. Bidirectional arrows indicate reciprocal influence rather than linear progression. For example, exploratory learning may increase cognitive and emotional demand, which in turn requires adaptive regulation; engagement both supports and is shaped by task design and learner capacity; and creativity emerges from the interaction of challenge, reflection, and prior knowledge rather than from any single instructional feature. The framework is therefore intended to guide interpretation and design reasoning, not to prescribe fixed instructional sequences.

At the center of the framework is the learner, emphasising that all components revolve around and support the learner’s development. At the base of the diagram is a foundational arrow labelled *Brain-Based and Neurodevelopmental Principles Applied Across K–12 Education*, indicating that all elements of the framework are grounded in neuroscience and developmental psychology. To the right of the learner are *Cognitive Skills and Non-Cognitive Skills*, represented in a green box. These are described as domains that develop in parallel and synergistically, acknowledging the integrated nature of intellectual and emotional growth. Above the learner is a trio of learning processes (i.e., *Exploration, Engagement, Creativity*) that feed directly into the learner and skill development pathways, indicating their role in stimulating cognitive and non-cognitive capacities. Exploration, engagement, and creativity are included as catalytic learning processes rather than exhaustive educational outcomes. Engagement encompasses emotional and motivational processes, while creativity reflects generative recombination and cognitive flexibility. Critical thinking is treated as an emergent capability drawing on these processes alongside executive control and domain knowledge, and emotions are embedded within engagement and self-regulation rather than represented as a separate component.

On the lower left, *Dynamic Cognitive Load Management* is bidirectionally linked to the learner, representing an ongoing, responsive adjustment of mental demands to fit the learner’s context, needs, and abilities. Located in the upper left (i.e., *Individualised and Inclusive Approaches*), this component connects with both the *Learner* and *Dynamic Cognitive Load Management*, signifying the importance of tailored instruction and recognition of neurodiversity. It is also linked to the *Exploration, Engagement, and Creativity* processes, emphasising the need to adapt these experiences to each learner.

### 8.1. Integration of Cognitive and Non-Cognitive Skills

The framework brings together and acknowledges the importance of cognitive skills (i.e., attention, memory, and problem-solving) while also integrating non-cognitive skills, which, as previously discussed, are equally critical to long-term learning and personal development. These non-cognitive skills include emotional regulation, self-regulation and interpersonal skills. Managing one’s emotions is crucial for learning since emotional experiences are intertwined with cognitive processes, and emotional regulation facilitates better focus, decision-making, and interpersonal interactions [116]. Development of self-regulation skills has been shown to be associated with the development of executive functions such as goal setting, attention management, and impulse control [117]. Executive functions are important for developing student independence in managing their learning effectively via enhanced cognitive capacity and the ability to adapt to challenging or unfamiliar learning contexts. Lastly, the framework emphasises the importance of non-cognitive skills such as interpersonal skills, which include empathy, cooperation, communication, and conflict resolution, to help students thrive in social and academic settings; and also the need to provide as required collaborative learning environments, which are integral to developing these skills [118].

The proposed framework encourages the parallel development of both cognitive and non-cognitive dimensions, recognising that social-emotional skills, executive functions, and cognitive abilities contribute to a child’s overall learning capacity. Unlike CLT, which focuses on minimising cognitive load, the NIHLDF emphasises the development and enhancement of cognitive and brain reserves. Early-life learning experiences, especially those that promote critical thinking, problem-solving, and social engagement, enhance cognitive reserve. The NIHLDF posits that children should be exposed to enriching, stimulating environments that foster these reserves and, most notably, support lifelong cognitive adaptability and resilience.

### 8.2. Adaptive Learning Environments

Moving beyond uniform instruction, the NIHLDF challenges the rigid instructional strategies resulting from the application of CLT and advocates for an alternative adaptive learning environment, tailored to learners’ needs. Classroom environments provide a diversity of cognitive and experiential backgrounds among learners. Some students may possess more substantial cognitive reserves and better self-regulation skills, while others may need more support. In acknowledgement of this diversity, the construct addresses the concerns of uniform instruction and encourages instructional practices that are personalised, flexible, and responsive to the individual needs of each learner, emphasising the importance of the following:

1. *Exploration*: Children’s natural curiosity and tendency to explore should be harnessed as a means of discovery and to use and develop critical thinking and problem-solving skills. This exploratory behaviour, which is more pronounced in children than adults, helps students develop a nuanced understanding of their environment and enhances neural flexibility [15].
2. *Engagement*: Learning environments should be stimulating, engaging, and aligned with real-world contexts where possible. Emotional and social engagement in learning tasks promotes deeper cognitive processing, intrinsic motivation, critical thinking, and long-term retention [90]. Recent findings also suggest that combined physical and cognitive scenarios maximise the engagement of participants and also their cognitive performance when compared with a single cognitive scenario [119].
3. *Creativity*: An environment with moderate levels of extraneous information and distraction can stimulate creativity and cognitive flexibility [120]. Creative thinking and cognitive flexibility are essential for dealing with complex, real-world problems that require adaptive thinking.

### 8.3. Recognising Diverse Cognitive Capacities and Individualised Approaches

In place of CLT’s emphasis on a universalisable instructional approach, the NIHLDF recognises individual neurocognitive differences. For example, cognitive load, rather than being managed uniformly, is understood as an individualised construct that varies substantially between learners, making it difficult to regulate effectively using a single, standardised approach for all students. Hence, the proposed framework emphasises the importance of personalised learning experiences based on the learner’s unique cognitive abilities, developmental stage, and socio-cultural background. Students with higher cognitive reserves might thrive with more complex tasks that challenge their working memory and executive functions. However, learners with lower reserves or learning difficulties can benefit from structured, scaffolded support that attends to their developing cognitive, emotional, and self-regulatory skills.

### 8.4. Dynamic, Flexible Cognitive Load Management: Emphasising Metacognition and Reflection

The NIHLDF recognises that cognitive load is not a fixed quantity, as CLT suggests, but rather a dynamic and flexible process, using a more fluid approach to managing cognitive demands, integrating cognitive and emotional challenges into the learning process. In the classroom, the NIHLDF encourages the inclusion of tasks that stimulate neural adaptability, promote creativity, and challenge students in ways that align with their developmental needs. For example (and accepting CLT working memory categories for the sake of this argument), regarding intrinsic load, the task’s inherent complexity can be managed by adapting tasks to the learner’s prior knowledge and abilities. Extraneous load can also be used strategically to foster creativity and cognitive flexibility rather than being viewed as information or distractions that may hinder learning. Lastly, germane load (i.e., cognitive resources dedicated to learning) should be maximised by ensuring the learner is engaged, motivated, and equipped with the tools to effectively self-regulate. Metacognitive strategies are also important as they foster self-regulation and long-term learning success. Teaching students to be aware of their thinking processes and providing them with tools for reflection (e.g., learning journals or exit tickets), self-monitoring, and adaptive problem-solving will allow them to better manage cognitive and emotional resources. This approach will foster resilience, adaptability, and lifelong learning skills.

## 9. Limitations and Educational Implications

To demonstrate further the conceptual distinctions between CLT and the proposed NIHLDF, Table 1 provides a comparative synthesis across key theoretical, developmental, and instructional dimensions. This comparison highlights both the limitations of traditional load-minimisation approaches and the broader educational implications of adopting a neurodevelopmentally informed, holistic perspective.

The NIHLDF is a conceptual, hypothesis-generating framework rather than a validated causal model. While grounded in empirical literature, it does not claim direct instructional prescriptions from neuroscience. Its constructs require further operationalisation and empirical testing in authentic classroom contexts, and its utility will ultimately depend on systematic evaluation across diverse educational settings. Indeed, future research should examine the NIHLDF using classroom-based experimental and longitudinal designs, including comparisons of uniform load-minimisation approaches with instructional sequences that integrate scaffolded challenge, reflection, and self-regulatory support. Mixed-method studies combining learning outcomes with behavioural indicators of engagement and regulation would be particularly valuable.

In practical terms, the NIHLDF suggests balancing periods of structured guidance with scaffolded challenge, reflection, and social interaction. Lessons may combine explicit instruction with generative problem-solving tasks, metacognitive reflection prompts, and collaborative learning experiences that support both content mastery and the development of self-regulatory capacity. This approach positions instructional design not as the minimisation of cognitive demand, but as the deliberate orchestration of learning experiences that simultaneously support knowledge acquisition, executive functioning, and socio-emotional development across diverse learners and developmental stages.

In the classroom, depending on Grade, subject, and student stage of development, pedagogical practices may include the use of worked examples followed by open-ended transfer tasks, structured peer discourse, reflective routines (e.g., learning journals, self-explanation prompts), and goal-setting activities. Assessment processes may transition from teacher-centred to student-centred, formative, process-oriented methodologies (e.g., portfolios, iterative drafts, and project-based learning) that reflect learners’ evolving metacognitive awareness, perseverance, resilience, and adaptive problem-solving skills. Classroom learning activities can be created to incorporate opportunities for productive struggle, collaborative inquiry, and exploratory play, particularly in the early stages of development when neural plasticity is heightened. At the curricular level, teaching and learning sequences can be structured to support conceptual development while also enhancing students’ executive functions, emotional regulation, and interpersonal skills, thereby reinforcing the framework’s focus on long-term adaptability rather than short-term instructional efficacy.

## 10. Conclusions

While CLT has influenced instructional design, there are significant limitations when considering meeting a range of student-specific learning needs and current research in neuroscience. The theory’s emphasis on managing cognitive load does not sufficiently address the development of interpersonal, emotional, and self-regulatory skills integral to effective, long-term learning and personal growth. Neuroscientific research highlights the value of these non-cognitive skills, demonstrating that learning environments that incorporate social, emotional, and self-regulatory elements foster cognitive and affective development [121,122].

By presenting the NIHLDF as an illustrative construct, this paper invites comparison with other approaches and encourages further research. The NIHLDF moves beyond the constraints of CLT by recognising the interconnectedness of learning’s cognitive, emotional, social, and self-regulatory aspects. It emphasises the need for enriched learning environments that support the development of cognitive and non-cognitive skills, adaptive approaches to cognitive load, and the importance of individualised instruction that accounts for the full range of developmental needs and capacities. The NIHLDF aims to create a more comprehensive, flexible, and practical approach to childhood learning and development by integrating insights from the fields of neuroscience, health sciences and educational psychology. By integrating the knowledge from these three fields, the NIHLDF encourages development toward a more holistic, flexible, and practical approach to the learning and development of children and young people. Moving beyond CLT, learning can be realised as a dynamic, multifield, and essentially social process that engages the whole child. This calls for a shift away from the narrow focus on content efficiency and cognitive load management to an expanded perspective, embracing the complexity of human development, in the creation of learning environments that foster the full range of human potential for not just cognitive growth, but also emotional well-being, social competence, and resilient, adaptable, lifelong learners prepared to successfully navigate their ever-changing world. To further this aim, future empirical research is important, particularly studies that evaluate the NIHLDF in varied educational environments. This effort would affirm its practical utility and enhance its contribution to educational theory and practice.

## Figures and Tables

**Table 1:** Comparative analysis of CLT and NIHLDF.

Dimension	CLT	NIHLDF
Theoretical Foundation	Late 20th-century cognitivist psychology; focuses on optimising instructional efficiency for novice learners acquiring well-structured knowledge.	Contemporary neuroscience, developmental psychology, and educational research; emphasises learning as a dynamic, distributed process across neural networks.
View of Working Memory	Relatively fixed-capacity system with strict limits; conceptualised as a cognitive ‘bottleneck’.	Dynamic, distributed neural network that is adaptable and context-sensitive; capacity is malleable through training and experience.
Cognitive Load Categories	Rigid distinction between intrinsic, extraneous, and germane load; treated as separable constructs.	Recognises cognitive processes as highly interdependent; load types dynamically interact and share limited attentional resources.
Primary Instructional Focus	Content delivery and schema acquisition; minimising cognitive overload.	Holistic development integrating cognitive, emotional, social, and self-regulatory competencies.
Emotional and Affective Factors	Largely peripheral to the theory; not systematically integrated into instructional design.	Central to learning; emotional engagement facilitates attention, memory consolidation, and motivation.
Social and Interpersonal Skills	Not explicitly addressed; focus remains on individual cognitive processing.	Integral component; social interaction strengthens neural networks and supports collaborative learning.
Self-Regulation and Executive Functions	Limited emphasis; cognitive load managed externally through instructional design.	Core focus; explicitly develops metacognition, attention management, planning, and adaptive problem-solving.
Approach to Individual Differences	Tends toward uniform instructional strategies applicable across learners.	Emphasises personalised, flexible instruction responsive to cognitive reserves, developmental stage, and background.
View of Cognitive and Brain Reserve	Not explicitly considered in instructional design.	Central construct; enriched environments and early-life experiences enhance neural efficiency and lifelong cognitive adaptability.
Role of Challenge and Difficulty	Emphasizes reducing extraneous load and simplifying content to prevent overload.	Values developmentally appropriate challenge; moderate difficulty promotes neural plasticity, creativity, and resilience.
Learning Environment Design	Structured, controlled environments that minimise distractions and extraneous information.	Enriched, stimulating environments that balance guidance with exploration, engagement, and creativity.
Developmental Considerations	Limited attention to developmental stage; primarily designed for novice learners.	Explicitly accounts for neurodevelopmental trajectories across K–12; recognises childhood as a period of heightened plasticity.
Default Mode Network (DMN)	Not considered; emphasis on continuous task engagement.	Acknowledges DMN’s role in memory consolidation, creative thinking, and cognitive flexibility.
Long-term Outcomes	Efficient knowledge acquisition and schema development.	Cognitive and brain reserve development, lifelong learning capacity, resilience, and adaptive expertise.

Note: CLT = Cognitive Load Theory; NIHLDF = Neurodevelopmental Informed Holistic Learning and Development Framework; DMN = Default Mode Network.

## Data Availability

No new data were created or analyzed in this study. Data sharing is not applicable to this article.

## Abbreviations

The following abbreviations are used in this manuscript:

ACC	Anterior Cingulate Cortex
CLT	Cognitive Load Theory
DMN	Default Mode Network
fMRI	Functional Magnetic Resonance Imaging
NIHLDF	Neurodevelopmental Informed Holistic Learning and Development Framework
PFC	Prefrontal Cortex
SEL	Social and Emotional Learning
